# Editorial: dose-dependent ZnO particle-induced acute phase response in humans warrants re-evaluation of occupational exposure limits for metal oxides

**DOI:** 10.1186/s12989-018-0247-3

**Published:** 2018-02-12

**Authors:** Ulla Vogel, Flemming R. Cassee

**Affiliations:** 1 National Research Centre for the Working Environment, Copenhagen, Denmark; 2 Department of Micro- and Nanotechnology, Technical University of Denmark, Kgs. Lyngby, Denmark; 3 National Institute for Public Health and the Environment (RIVM), Bilthoven, The Netherlands; 4 Institute for Risk Assessment Sciences, Utrecht University, Utrecht, The Netherlands

## Abstract

Epidemiological studies link inhalation of particles to increased risk of cardiovascular disease. Inhaled particles may induce cardiovascular disease by several different mechanisms including translocation of particles to systemic circulation, activation of airway sensory nerves resulting in autonomic imbalance and particle-induced pulmonary inflammation and acute phase response.

The acute phase response is the systemic response to acute and chronic inflammatory states caused by for example bacterial infection, virus infection, trauma and infarction. It is characterized by differential expression of ca. 50 different acute phase proteins including C-reactive protein and Serum amyloid A, which are the most differentially up-regulated acute phase response proteins. Blood levels of these two acute phase proteins are closely associated with risk of cardiovascular disease in epidemiological studies and SAA has been causally related to the formation of plaques in the aorta in animal studies.

In a recent paper in Particle and Fibre Toxicology, Christian Monsé et al. provide evidence that inhalation of ZnO nanoparticles induces dose-dependent acute phase response in humans at dose levels well below the current mass-based occupational exposure limits in a number of countries including Germany, The Netherlands, UK, Sweden, Denmark and the US.

Given the evidence suggesting a causal relationship between increased levels of serum amyloid A and atherosclerosis, the current results call for a re-evaluation of occupational exposure limits for a number of particle exposures including ZnO taking induction of acute phase response into account. Furthermore, it underscores cardiovascular disease as an occupational disease.

Epidemiological studies link inhalation of particles to increased risk of cardiovascular disease [1]. Inhaled particles may induce cardiovascular disease by several different mechanisms including translocation of particles to systemic circulation, activation of airway sensory nerves resulting in autonomic imbalance and particle-induced pulmonary inflammation and acute phase response [2].

In a recent paper in Particle and Fibre Toxicology, Christian Monsé et al. [3] provide evidence that inhalation of ZnO nanoparticles induces dose-dependent acute phase response in humans at dose levels well below the current mass-based occupational exposure limits in some countries including Denmark and the US. The acute phase response is the systemic response to acute and chronic inflammatory states caused by for example bacterial infection, virus infection, trauma and infarction [4]. It is characterized by differential expression of ca. 50 different acute phase proteins including C-reactive protein (CRP) and Serum amyloid A (SAA) which are the most differentially up-regulated acute phase response proteins. Blood levels of these two acute phase proteins are closely associated with each other and with risk of cardiovascular disease in epidemiological studies [5–7].

The presence of SAA has been causally related to the formation of plaques in the aorta. Overexpression of SAA leads to increased plaque progression and inhibition of SAA synthesis leads to lowered plaque progression in APOE −/−mouse models [8, 9]. SAA plays an important role in cholesterol transport. Under homeostasis, HDL facilities cholesterol transport from peripheral macrophages to the liver. During the acute phase response, SAA is incorporated into HDL lipoproteins, where it alters the cholesterol flow, causing peripheral macrophages to accumulate cholesterol and turn into foam cells, thus causing plaque progression and atherosclerosis [10, 11]. SAA has also been suggested to contribute to endothelial dysfunction, promote thrombosis, and enhance leukocyte trafficking and activation [12]. On the other hand, there is little evidence that CRP is causally related to risk of cardiovascular disease. Mendelian randomization studies showed that genetically determined differences in CRP levels were not associated with risk of cardiovascular disease [13, 14]. Overall, this suggests that the acute phase response plays a causal role in atherosclerosis. Even modest changes in blood levels of SAA and CRP are associated with risk of cardiovascular disease in humans. A 5-fold increase in SAA levels was associated with 3-fold increased risk for cardiovascular events defined as death from coronary heart disease, nonfatal myocardial infarction or stroke, or the need for coronary-revascularization procedures in the Nurses’ Health Cohort [5].

In mice, inhalation and pulmonary exposure to a number of different nanoparticles and other particles has been shown to induce long-lasting time and dose-dependent pulmonary acute phase response [15, 16]. The pulmonary acute phase response was shown to be proportional to the total surface area of the pulmonary deposited particles [15], neutrophil influx in the bronchoalveolar lavage fluid [17] and to blood levels of SAA [18].

In humans, blood levels of acute phase proteins have been shown to be associated with exposure to various particle exposures including organic dust [19], dust from a paper mill [20], welding fumes [21, 22] and fumes developed during firing of small weapons [23]. This suggests that SAA and CRP levels may constitute sensitive and robust biomarkers of particle-induced cardiovascular disease. Christian Monsé et al. show dose-dependent acute phase response following a 4-h exposure to ZnO nanoparticles at 0.5–2 mg/m^3^. SAA levels were statistically significantly increased ca. 5 fold at 1 mg/m^3^ compared to sham exposure. These levels are well below the occupational exposure limit for ZnO which is 5–10 mg/m^3^ ZnO for UK, Germany, The Netherlands, Sweden, Denmark and the US (https://www.osha.gov/dts/chemicalsampling/data/CH_277005.html) [24]. Moreover, exposure to welding fumes with Zn and Cu also induced acute phase response in human volunteers [22].

Given the evidence suggesting a causal relationship between increased SAA levels and atherosclerosis, the current results call for a re-evaluation of occupational exposure limits for a number of particle exposures including ZnO taking induction of acute phase response into account. Furthermore, it underscores cardiovascular disease as an occupational disease.
